# Fatigue, sleepiness and depression in multiple sclerosis: defining the overlaps for a better phenotyping

**DOI:** 10.1007/s00415-022-11143-6

**Published:** 2022-05-04

**Authors:** Davide Sparasci, Claudio Gobbi, Anna Castelnovo, Gianna Carla Riccitelli, Giulio Disanto, Chiara Zecca, Mauro Manconi

**Affiliations:** 1 Sleep Medicine Unit, Neurocenter of Southern Switzerland, Ospedale Civico, Lugano, Switzerland; 2 Multiple Sclerosis Center, Neurocenter of Southern Switzerland, Ospedale Regionale di Lugano, EOC, Lugano, Switzerland; 3 University Hospital of Psychiatry and Psychotherapy, University of Bern, Bern, Switzerland; 4 Faculty of Biomedical Sciences, Università della Svizzera Italiana, Lugano, Switzerland; 5 Neuropsychology and Behavioural Neurology Research Unit, Neurocenter of Southern Switzerland, Ospedale Civico, Lugano, Switzerland

**Keywords:** Multiple sclerosis, Sleep, Fatigue, Depression, Maintenance of wakefulness test

## Abstract

**Background and objectives:**

To define the boundaries and the overlaps between fatigue, sleepiness and depression in patients with multiple sclerosis (MS) by using different tools for each dimension, including instrumental sleep analysis.

**Methods:**

In this cross-sectional, observational study, 71 MS patients (males/females: 20/51; mean age: 48.9 ± 10.5 years) filled in clinical questionnaires and performed polysomnography followed by maintenance of wakefulness test (MWT). Frequency and reciprocal overlap of sleepiness, fatigue and depression in MS were expressed by Eulero-Venn diagrams; standard multiple regression was used to assess the ability of symptoms to predict each other.

**Results:**

There was a high percentage of fatigued (70%), somnolent (45%) and depressed (27%) patients. Fatigue had the strongest overlap and correlated with both depression (beta: 0.52, *p* < 0.001) and sleepiness (beta: 0.74, *p* < 0.001). Somnolence and depression were nearly always accompanied by fatigue and were well differentiated from each other by MWT. Four MS subgroups were identified that had: (1) fatigue only; (2) fatigue and sleepiness (3) fatigue and depression; (4) fatigue, sleepiness and depression.

**Discussion:**

The subjective and objective tools are not able to clearly distinguish fatigue from sleepiness and depression, while only a test of vigilance can be helpful in separating somnolence and depression from each other.

## Introduction

Multiple Sclerosis (MS) is a chronic inflammatory disease of the central nervous system (CNS), causing a wide range of symptoms as consequence of an unpredictable focal as well as diffuse CNS involvement [1]. Beyond the focal neurologic deficits, related to the site of lesions, the quality of life of patients with MS can be highly compromised by systemic complaints like fatigue, depression [2] and excessive daytime sleepiness (EDS) [3].

Fatigue affects between 60 and 90% of patients with MS, being sometimes the most debilitating symptom [4]. Depression is the most common psychiatric symptom in MS, with a prevalence up to 50% of patients. However, fatigue is also included among the diagnostic symptoms of the major depressive disorder. On the other hand, fatigue may cause depression by reducing vitality and daytime activities [5].

EDS refers to an abnormal likelihood of dozing during normal waking hours and is a complaint endorsed by 19–34% of MS patients representing a potential confounding or overlapping factor [6–8]. Sleep disorders such as insomnia and restless legs syndrome are also very common in MS [9], and may result in or exacerbate EDS, but also fatigue and depression [10]. Moreover, sleep quality in terms of duration, efficiency, and macrostructure is decreased in MS patients compared to healthy controls [11].

Due to the high frequency of fatigue, depression, and EDS in MS and to their possible comorbid overlap, it is very difficult for clinicians to disentangle between these and finally to tailor the most proper treatment. Furthermore, patients may subjectively misinterpret their symptom, confounding one for another. In light of this, it is not surprising that the use of stimulants and antidepressants to contrast fatigue in MS [12–14] has shown controversial efficacy. In this complex scenario the definition of clinical phenotypes may guide the therapeutic choice and would prepare the ground for future trials.

In order to identify clinical MS subgroups and better personalize the management of fatigue, we defined the boundaries and the overlaps between fatigue, depression and EDS. Two different tools for each symptom were employed, including the MWT to test vigilance performance. We were interested in establishing whether clinical questionnaires could discriminate between the three symptoms, and the potential role of the MWT in the diagnostic algorithm of fatigued patients with MS.

## Methods

### Participants

A cross–sectional, observational, instrumental, single-center study in a sample of 71 patients older than 18 and affected by MS according to McDonald criteria [15] or clinically isolated syndromes (CIS) [16] was carried out at the Neurocenter of Southern Switzerland in Lugano. Additional inclusion criterion was an Expanded Disability Status Scale (EDSS) score < 7.0 (range 0–10). Exclusion criteria were the following: Mini Mental Status Examination (MMSE) score lower than 24; recent (within the past 3 months) clinical MS relapse; radiologically isolated syndrome (RIS); history of drug and/or alcohol abuse; any serious general medical condition such as decompensated cardiopulmonary disease, cancer or decompensated renal failure, as well as any major neurological condition other than MS that could interfere with the correct execution of the study design. Based on its clinical course, MS was classified as primary progressive, secondary progressive, or relapsing remitting.

### Study design

#### Self-report measures

At the screening visit, patients were interviewed concerning their medical history and received a complete clinical and neurological examination including the EDSS evaluation and a MMSE assessment. All patients filled in the following self-administered validated questionnaires: Epworth Sleepiness Scale (ESS) (range: 0–24; cutoff for normality: ≤ 10) [17], Beck Depression Inventory—Second Edition (BDI-II) (range: 0–63; cutoff for normality: ≤ 9; cutoff for moderate depression ≥ 20) [18, 19], Montgomery-Åsberg Depression Rating Scale (MADRS) (range: 0–60; cutoff for normality: ≤ 13) [20], Fatigue Severity Scale (FSS) (range: 9–63; cutoff for normality: < 36) [21], Modified Fatigue Impact Scale (MFIS) (range: 0–84; cutoff for normality: < 38) [22], Multiple Sclerosis Quality of Life 54 (MSQoL-54) (range: 0–00) [23], Pittsburgh Sleep Quality Index (PSQI) (range: 0–21; cutoff for normality: < 5) [24].

#### Polysomnography and maintenance of wakefulness test

Within 1 week from the screening visit, participants underwent a full night polysomnography (PSG) by a portable device (Embletta ST + Proxy), and a Maintenance of Wakefulness Test (MWT) the following day.

The PSG montage included the following: EEG, electrooculogram, electromyogram (EMG) of chin and both tibialis anterior muscles, electrocardiogram, body position; oro-nasal airflow (nasal pressure cannula), thoracic and abdominal movements, and oxygen saturation.

The MWT explores objectively the ability to remain awake in sleep-promoting environmental conditions [25]. The montage included the following signals: EEG (6 channels); electrooculogram (2 channels); electromyogram (EMG) of the submentalis muscle. The MWT was carried out in a dim room, with patients instructed to stay awake as long as possible. Four sessions were recorded every two hours, each lasting until the patient fell asleep or up to 40 min. Sleep latency was assessed in each of the four recordings based on the first epoch of sleep. The final result consisted of the mean of sleep latency calculated over the four sessions. Two pathological thresholds were defined, one corresponding to low-moderate severity at 30 min, and at 20 min for severe cases [26].

### Statistical analysis

Descriptive statistics were used in the first instance. Correlations were subsequently assessed by means of the Pearson’s correlation coefficient. Following the indications by Cohen, we considered correlations 0.10, 0.30, and 0.50 as corresponding to small, medium, and large sizes, respectively [27]. Standard multiple regression was used to assess the ability of sleepiness, fatigue and depression to predict each other, after preliminary analyses to ensure no violation of the assumptions of normality, linearity, multicollinearity. Primary outcomes were the scores of FSS, MFIS, ESS, BDI, MADRS and the mean sleep latency at the MWT. Finally, a one-way between-groups analysis of variance (ANOVA), with post-hoc comparisons using the Tukey–Kramer test (unequal sample sizes) and Bonferroni correction to avoid Type I error, was conducted to compare clinical and paraclinical parameters among the 4 different phenotypes of MS patients with fatigue. The level for statistical significance was set at *p* < 0.05.

## Results

Mean age of patients was of 48.9 ± 10.5 years. Fifty-one (71.8%) were women, mean disease duration was 10.6 ± 8.1 years, 61 (85.9%) patients had relapsing remitting MS, and 9 (12.7%) had a moderate–severe disability (EDSS ≥ 4). Table 1 reports anthropometrics and clinical data.

**Table 1:** Demographic and clinical parameters

MS (*n* = 71)		% pathological
Age, mean, SD	48.9 ± 10.5	
Male, *n*, %	20 (28.2%)	
Female, *n*, %	47 (71.8%)	
CIS, *n*, %	5 (7.0%)	
RR-MS, *n*, %	61 (85.9%)	
SP-MS, *n*, %	3 (4.2%)	
PP-MS, *n*, %	2 (2.8%)	
Disease duration (years), mean, SD	10.6 ± 8.1	
EDSS, mean, SD	2.6 ± 1.4	
MSQoL-54, mean, SD	60.0 ± 21.3	
FSS, mean, SD	41.3 ± 17.0	69
MFIS, mean, SD	44.4 ± 21.7	70.4
ESS, mean, SD	9.2 ± 4.8	45
MWT mean SL, SD	34.0 ± 8.8	11.2^a^
BDI-II, mean, SD	14.1 ± 11.1	26.8
MADRS, mean, SD	9.9 ± 6.0	31
PSQI, mean, SD	9.2 ± 4.0	91.5

MS: multiple sclerosis; CIS: clinical isolated syndrome; RR-MS: relapsing–remitting multiple sclerosis; SP-MS: secondary progressive multiple sclerosis; PPMS: primary progressive multiple sclerosis; EDSS: expanded disability status scale; MSQoL-54: multiple sclerosis quality of life 54; ESS: Epworth Sleepiness Scale; MWT: maintenance of wakefulness test; SL: sleep latency; FSS: Fatigue Severity Scale; MFIS: modified fatigue impact scale; BDI-II: Beck Depression Inventory—Second Edition; MADRS: Montgomery–Åsberg Depression Rating Scale; PSQI: Pittsburgh sleep quality index; SD: standard deviation

^a^20 minutes cutoff (25.4% for 30 min cutoff)

Bold values: statistically significant (*p* < 0.05)

Forty-nine (69%) and 59 (70.4%) patients were fatigued according to the FSS and MFIS respectively (mean FSS score: 41.3 ± 17.0; mean MFIS score 44.4 ± 21.7).

Nineteen (26.8%) and 22 (31%) had an at least moderate depression according to BDI-II and MADRS scores, respectively.

Sleep quality was reduced in 64 (91.5%) patients (PSQI ≥ 5, mean PSQI score 9.2 ± 4.0).

Subjective EDS, as assessed by ESS (ESS > 10), affected 32 (45%) patients. At the MWT, 8 (11.2%) patients showed a mean sleep latency < 20 min (severely sleepy), 18 (25.4%) had the pathological threshold at < 30 min (moderately sleepy). Among patients with an ESS score > 10 points, 7 (9.4%) had a mean sleep latency < 20 min, 20 (28.1%) < 30 min, and the remaining 71.9% resulted normal at the MWT.

### Measures of fatigue, sleepiness and depression

**Fig. 1:**
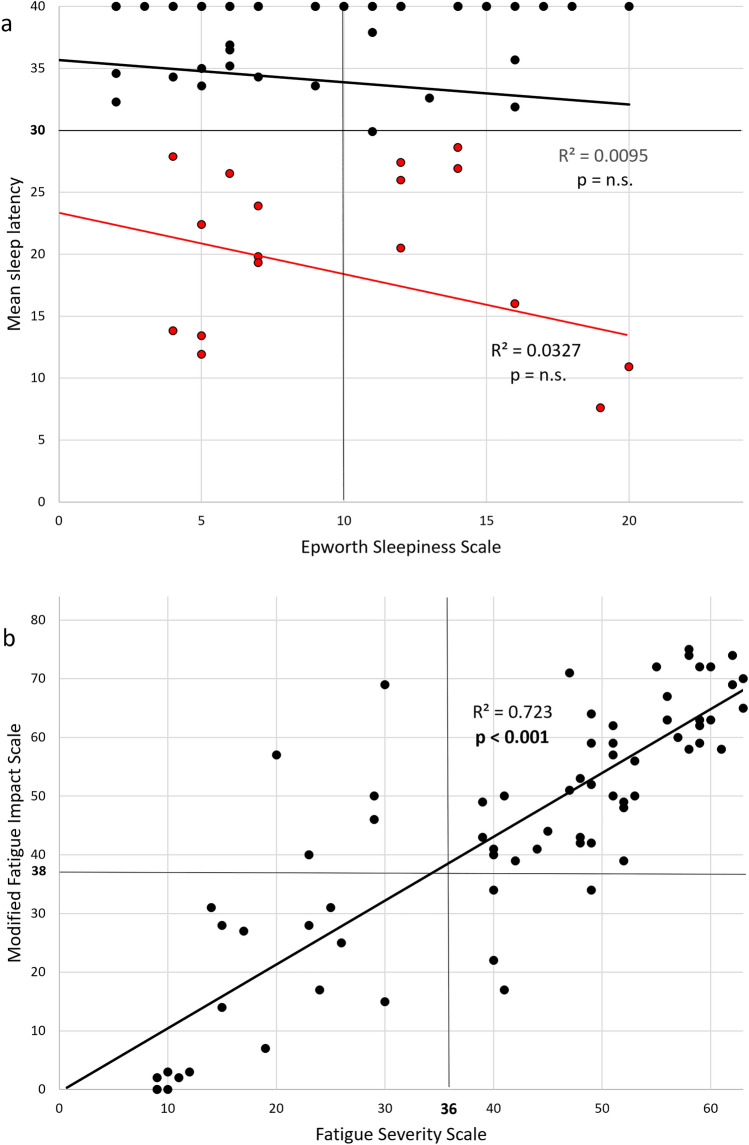
Linear correlations between Epworth Sleepiness Scale (ESS) and mean sleep latency at the Maintenance of Wakefulness Test (MWT) (**a**) (black line). Linear correlation between Fatigue Severity Scale and Modified Impact Fatigue Scale (**b**). The red line illustrates the correlation between ESS and MWT only in patients with a pathological MWT (red spots)

There was a strong correspondence between the two measures of fatigue FSS score and MFIS (*r* = 0.850, *p* < 0.001) (Fig. 1b).

The two measures of sleepiness ESS score and sleep latency at the MWT were not correlated (*r* = 0.097, p not significant) (Fig. 1a).

There was a significant linear correlation between the two measures of depression BDI-II and MADRS (*r* = 0.606, *p* < 0.001) (Table 2).

**Table 2:** Correlations between sleepiness (ESS, MWT), fatigue (FSS, MFIS), and depression (BDI-II, MADRS) assessed by standard multiple regression

	FSS		Multiple regression
	vs.	Beta	*p* <
ESS	BDI-II	0.32	**0.001**
BDI-II	ESS	0.59	**0.001**
MADRS	ESS	0.42	**0.001**
MWT	BDI-II		n.s.
MWT + ESS	BDI-II		n.s.

	ESS		Multiple regression
	vs.	Beta	*p* <
BDI-II	FSS		n.s.
MADRS	FSS		n.s.
FSS	BDI-II	0.52	**0.001**
MFIS	BDI-II	0.51	**0.001**

	BDI-II		Multiple regression
	vs.	Beta	*p* <
ESS	FSS		n.s.
MWT	FSS		n.s.
FSS	ESS	0.70	**0.001**
MFIS	ESS	0.74	**0.001**

ESS: Epworth Sleepiness Scale; MWT: maintenance of wakefulness test; FSS: Fatigue Severity Scale; MFIS: modified fatigue impact scale; BDI-II: Beck Depression Inventory—Second Edition; MADRAS: Montgomery-Åsberg Depression Rating Scale; vs.: versus

Bold values: statistically significant (*p* < 0.05)

### Overlap between fatigue, sleepiness, and depression

**Fig. 2:**
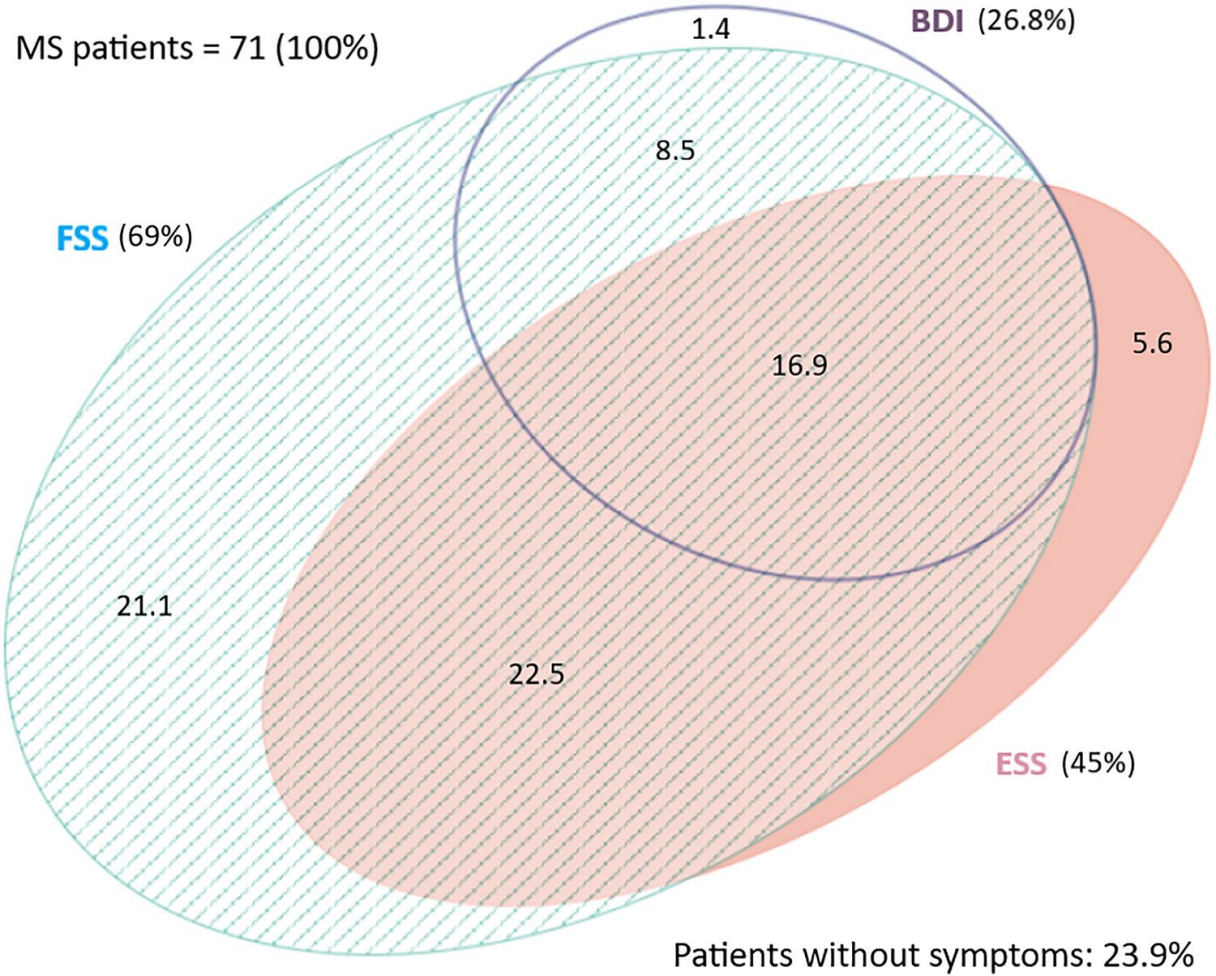
Frequency and reciprocal overlap of sleepiness, fatigue and depression in MS, as expressed by Eulero-Venn diagrams. MS: multiple sclerosis; BDI-II: Beck Depression Inventory—Second Edition; ESS: Epworth Sleepiness Scale; FSS: Fatigue Severity Scale. All the digits are expressed in percentages

As illustrated in Fig. 2a, there was a consistent overlap between depression, fatigue and subjective sleepiness.

**Fig. 3:**
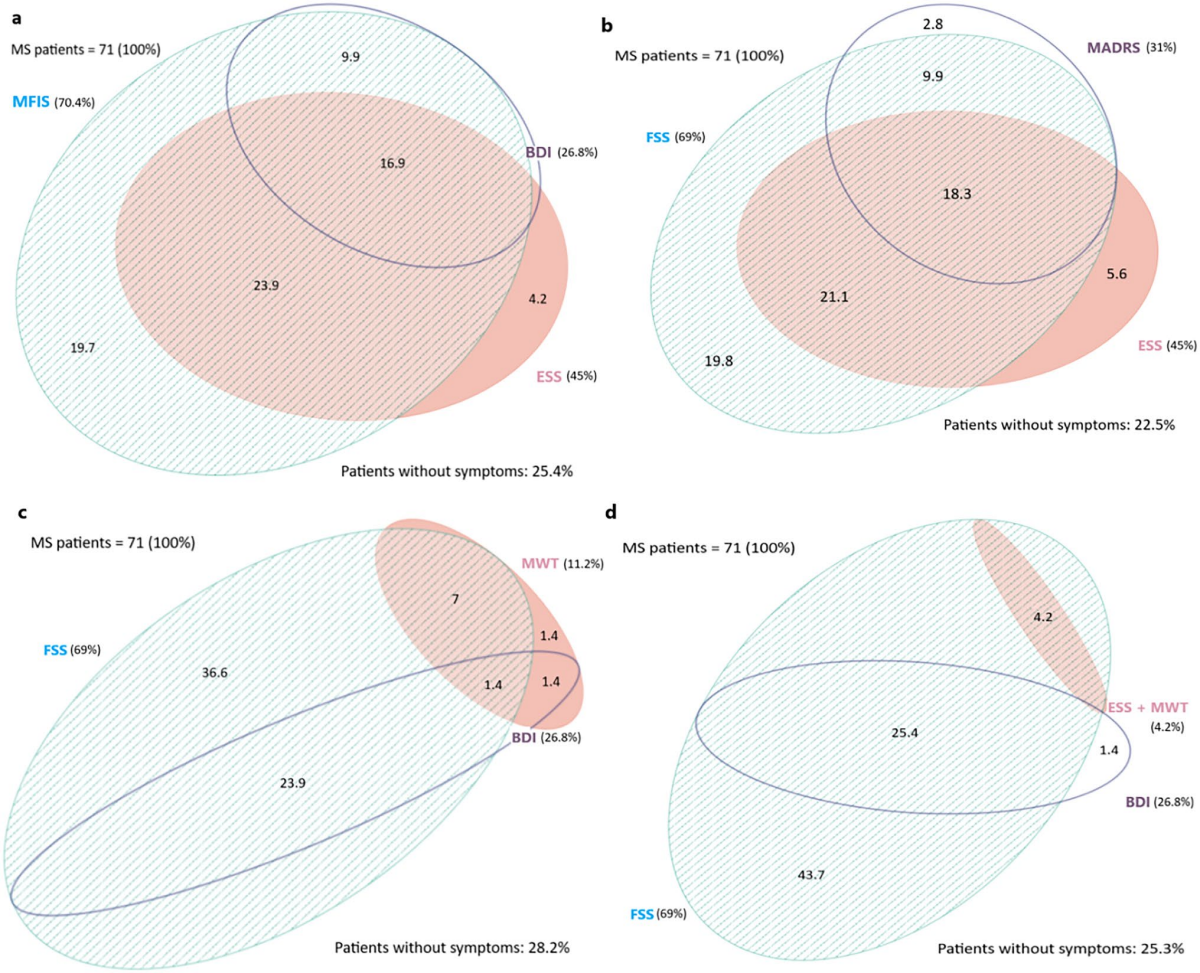
Frequency and reciprocal overlap of sleepiness, fatigue, and depression in MS, as expressed by Eulero-Venn diagrams. MS: multiple sclerosis; MFIS: modified fatigue impact scale; BDI-II: Beck Depression Inventory—Second Edition; MADRS: Montgomery–Åsberg Depression Rating Scale; ESS: Epworth Sleepiness Scale; FSS: Fatigue Severity Scale; MWT: maintenance of wakefulness test; All the digits are expressed in percentages

Almost all depressed patients (BDI-II ≥ 20) were also fatigued (94.8%) according to FSS, and 45 (63%) were subjectively drowsy according to ESS. Sixty-three percent of patients had both subjective EDS and fatigue. Less than one-third (28.1%) of patients presented only one out of fatigue, depression or sleepiness. The degree of overlap between fatigue and sleepiness was slightly reduced when defining sleepiness by means of pathological MWT (cut-off 20 min) compared to ESS (75% and 87.6% of drowsy patients with fatigue, respectively) (Fig. 2b). All patients with pathological scores at both ESS and MWT were fatigued, but none was depressed (Fig. 2c). The employ of MFIS instead of FSS as a measure of fatigue, did not change the overlap of the three symptoms (Fig. 2a vs. Fig. 3a, b).

### Correlation between sleepiness, fatigue and depression

Standard multiple regression was used to assess the ability of sleepiness and depression, as measured by ESS and BDI-II, to predict levels of fatigue (FSS). The total variance explained by the model as a whole was 56.3%. ESS and BDI-II were statistically significant, with BDI-II recording a higher beta value (beta = 0.59, *p* < 0.001) than ESS (beta = 0.32, *p* < 0.001). Very similar results were obtained for categorical BDI-II (cut off: 20 points), MADRS, and categorical ESS (cut off: 10 points).

The levels of fatigue, as measured by FSS or MFIS, were independently correlated to BDI-II (FSS: beta = 0.70, *p* < 0.001; MFIS: beta = 0.74, *p* < 0.001) and sleepiness (FSS: beta = 0.52, *p* < 0.001; MFIS: beta = 0.51, *p* < 0.001). Subjective drowsiness and depression, as measured with MADRS or BDI-II, were uncorrelated.

Sleep latency at MWT, alone or in combination with ESS, did not independently correlate to depression and fatigue (either FSS or MFIS), even if adopted as a categorical variable (cut off: either 20 or 30 min).

### Clinical subtypes

Fatigue as measured by either FSS or MFIS was the most overlapping symptom as 28 (87.5%) sleepy (ESS) and 18 (94.8%) depressed (BDI-II) patients also complained about fatigue, but not vice versa. Subjects with isolated depression or drowsiness were rare (1.4% and 5.6% respectively). We accordingly defined the following four main phenotypes of fatigued patients: (1) fatigue alone; (2) fatigue + sleepiness; (3) fatigue + depression; (4) fatigue + sleepiness + depression. These phenotypes did not differ in terms of age, disease duration, disability status, subjective sleep quality, sleep latency at the MWT, periodic limb movements during sleep, sleep-related breathing disorders, and PSG parameters. Patients belonging to *phenotype 4* had a worse physical quality of life than those belonging to *phenotype 1 or 2* (Table 3), even after applied Bonferroni correction.

**Table 3:** Comparison of clinical and paraclinical parameters between the four different phenotypes of MS patients with fatigue

	Phenotype 1 Fatigue only (*n* = 15)		Phenotype 2 Fatigue + EDS (*n* = 16)		Phenotype 3 Fatigue + depression (*n* = 6)		Phenotype 4 Fatigue + EDS + depression (*n* = 12)		One-way ANOVA
	Mean	SD	Mean	SD	Mean	SD	Mean	SD	*p* (between groups)
Age	51.20	10.94	49.06	12.30	49.50	7.15	47.92	12.07	0.96
MS duration from diagnosis	8.37	6.10	12.68	10.06	9.75	8.22	12.71	8.97	0.61
Expanded disability status scale	2.83	1.32	3.06	1.21	3.00	1.52	2.96	1.10	0.81
PSQI	10.10	4.07	8.75	3.17	12.7	2.80	10.78	4.41	0.27
MS quality of life physical	60.68*	16.05	55.93*	15.26	44.39	9.67	35.25*	10.55	** < 0.01**
MWT, min	33.15	7.50	33.59	10.59	35.30	7.39	33.97	8.56	0.94
TST, min	356.60	65.53	364.00	66.41	341.33	53.79	393.18	64.33	0.45
SOL, min	28.13	27.85	21.86	21.30	33.17	28.71	14.09	15.51	0.43
FRL, min	110.67	65.96	98.93	63.05	169.50	84.75	81.45	78.25	0.19
SS-h	19.13*	5.63	20.71	4.71	26.33*	4.89	19.45*	4.23	**0.03**^**†**^
AWN-h	5.40	2.53	6.07	2.06	8.00	2.45	6.82	2.93	0.15
SE, %	79.07	12.23	81.21	9.14	80.00	9.08	83.36	7.89	0.86
WASO, %	13.40	11.50	13.71	9.86	12.33	6.92	12.45	6.47	0.95
S1, %	9.20	3.63	11.36	4.85	13.33	5.13	9.91	3.02	0.29
S3, %	17.33	8.23	18.50	7.44	20.00	5.22	16.82	5.96	0.92
REM, %	17.07	8.04	17.07	6.07	14.83	7.20	19.82	6.82	0.61
PLMSI	24.47	41.79	9.07	17.28	28.17	35.71	16.82	20.35	0.60
RDI	6.71	11.88	9.91	18.46	7.00	9.06	6.00	4.63	0.95
T90	0.08	0.28	2.00	4.67	0.00	0.00	1.70	3.16	0.49
ODI	1.00	1.47	4.82	7.13	0.00	0.00	5.00	7.12	0.24

EDS: excessive daytime sleepiness; MS: multiple sclerosis; PSQI: Pittsburgh sleep quality index; SD: standard deviation; MWT: maintenance of wakefulness test; TST: total sleep time; SOL: sleep onset latency; FRL: first REM latency; SS-h: stage shift per hour; AWN-h: awakening index per hour; SE: sleep efficiency; WASO: wake after sleep onset; PLMSI: periodic limb movements during sleep index; RDI: respiratory disturbance index; T90: percentage of sleep time with an SpO_2_ < 90%; ODI: oxygen desaturation index

^*^Difference in mean scores statistically significant (*p* < 0.05) between two groups after post-hoc analysis (Tukey–Kramer Test)

^†^Not significant after Bonferroni correction

Bold values: statistically significant (*p* < 0.05)

## Discussion

This present study evaluated, by using different assessment tools, the overlaps and correlations between three critical systemic symptoms reported by patients with MS: fatigue, somnolence, and depression.

Each of these symptoms was investigated in our study by means of two different tools and diagnosed in case of pathological findings in at least one of these two. We found a high percentage of fatigued (70%), somnolent (45%), and depressed (27%) patients in our sample, according to FSS, ESS, and BDI-II, respectively. While the two questionnaires used to measure fatigue (FSS, MFIS) as well as the two used to measure depression (BDI-II, MADRAS) were correlated with a large size effect, MWT and ESS did not. As the Eulero-Venn diagrams illustrate (Fig. 2), the overlap between depression, fatigue and subjective sleepiness was very consistent. Fatigue overlapped the most and showed an independent correlation with the other two symptoms. Sleepiness and depression overlapped much less each other when MWT instead of ESS was used (Fig. 3b) and just in very few cases were not associated with fatigue.

In line with our study, the literature reports a prevalence of fatigue in MS patients between 60 and 90% [28–31]. Most studies show a frequency of depression in MS similar to ours, ranging between 5 and 60% (mean 27%) [32], but usually a lower prevalence of subjective sleepiness (19–34%) [6–8, 33].

Depressed MS patients develop fatigue 3.6 times more than non-depressed ones, according to Alarcia et al. findings [34]. In two distinct studies Beata LR et al. [35] showed a linear correlation between MADRS and FSS scores, confirming the findings of previous works [36, 37]. Differently, Vercoulen et al. [38] found no significant association between fatigue and depression in patients with MS. However, they employed a different scale for fatigue assessment (Checklist of Individual Strength) and recruited far more patients with PP-MS (38% vs. 2.8%). This is relevant, since a recent study [39] found a correlation between FSS and BDI-II in RR-MS but not in PP-MS.

In line with the present study, Stanton et al. [6] found that FSS and ESS scores were significantly, although weakly (*p* = 0.02), correlated. A relationship between fatigue and sleepiness in MS remains controversial, being supported by some studies [7, 33, 40, 41], but not others [8, 42–45]. For instance, Kaynak et al. [8] using both subjective (ESS) and objective (sleep latency at the MSLT) measures of sleepiness, showed conflicting results: FSS did not correlate with ESS nor sleep latency, while Fatigue Impact Scale did so. Remarkably, 53% of drowsy and fatigued patients with MS have been diagnosed with central hypersomnia by means of 24-h PSG and MSLT [40].

Three studies [35, 46, 47] concluded for a positive correlation between BDI-II and ESS; nevertheless, they did not consider fatigue as a confounder. In fact, we did not find the same association after a multiple regression analysis including fatigue as independent variable. In line with our results, Ghajarzadeh et al. performed multiple linear regression analyses including fatigue, sleepiness and depression, finding both ESS and BDI-II as predictors of fatigue (MFIS). No studies are available on the reciprocal overlap between the three symptoms. Only three small studies [8, 40, 46] (32, 37, 44 patients) included a vigilance test (MSLT), but none used the MWT. According to our findings, sleep latency did not correlate with fatigue or subjective sleepiness (ESS). The discrepancy between MWT and ESS is not surprising and described also in other disorders, such as OSAS and narcolepsy [48].

We believe the consistent overlap between fatigue, sleepiness, and depression might likely have one or more of the following explanations: (1) *comorbidity*: the three symptoms are frequently and truly concurrent; (2) *accuracy*: the assessments used are inadequate to differentiate among these three conditions; (3) *misunderstanding*: MS patients tend to confuse/misrecognize fatigue from drowsiness and depression, while better distinguishing between bad mood and sleepiness; (4) *dependency*: there is a causal relationship among the three symptoms, meaning that one favours the occurrence of another or both. For example, depressive symptoms can result in secondary fatigue, namely cognitive fatigue. The implementation of MWT reduces the “*misunderstanding*” hypothesis, being much less dependent from the subjective evaluation. Despite the use of different tools to identify the three symptoms, “*accuracy”* remains an issue, mainly in the assessment of fatigue rather than the two other dimensions. Finally, the border between “*comorbidity”* and “*dependency”* is blurred and both address to a real overlap, which remains the best hypothesis until proven otherwise.

**Fig. 4:**
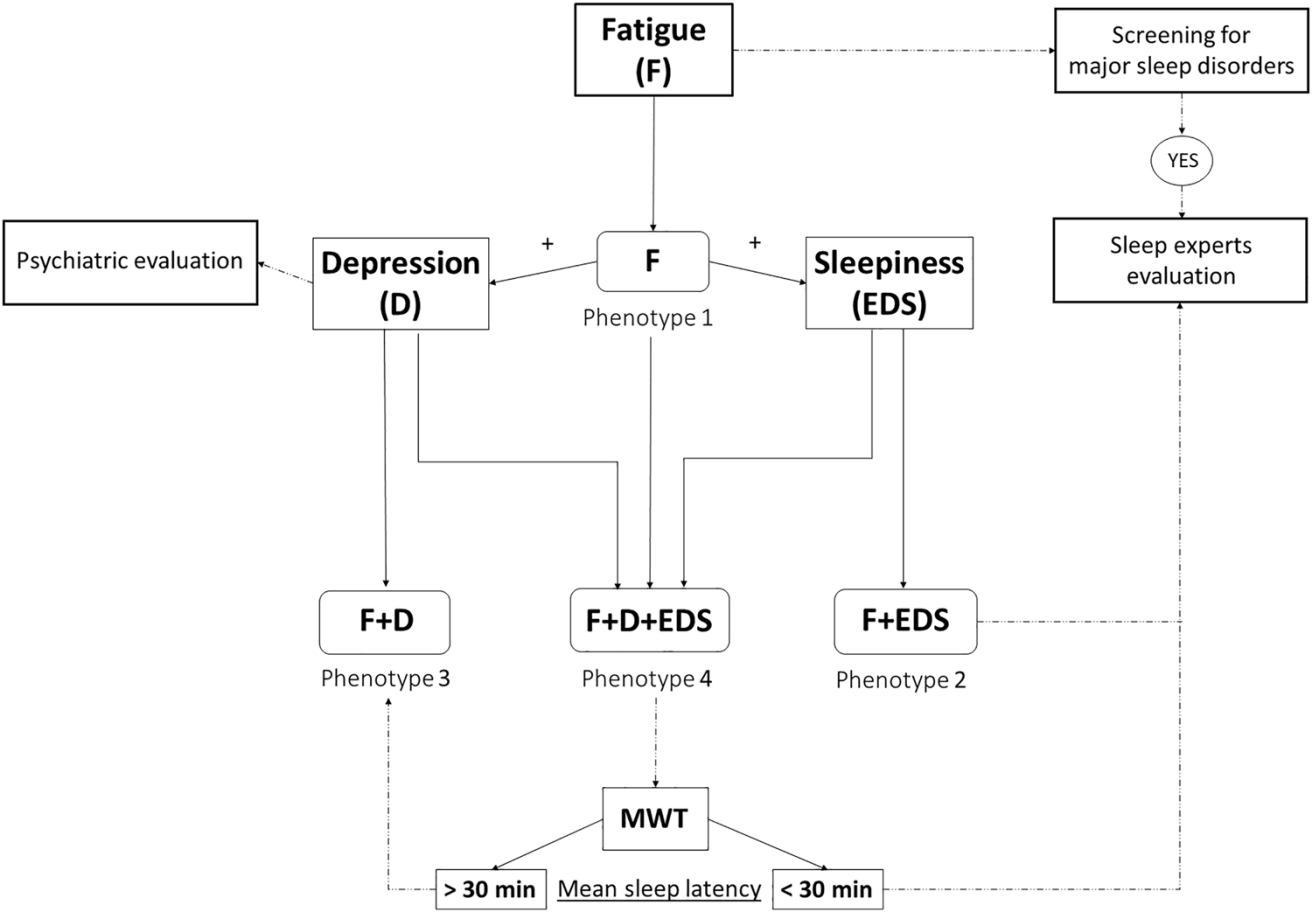
Diagnostic diagram to assess clinical phenotype in patients with multiple sclerosis suffering from fatigue. Whether a patient with MS complains of fatigue, clinical interview and questionnaires are advisable to exclude somnolence, bad mood, as well as major sleep disorders. A test of vigilance is recommended to better distinguish somnolence from depression if the two symptoms coexist (*phenotype 4*). Whether the patient is depressed, a psychiatric counselling is recommended. In case of drowsiness or pathological MWT, a sleep evaluation at the sleep lab is suggested to exclude specific sleep disorders. MWT: maintenance of wakefulness test (cutoff: sleep latency of 30 min); EDS: excessive daytime sleepiness; F: fatigue; D: depression; min: minutes

Our approach allows the identification of the following four main sub-groups of fatigued patients: (1) those with fatigue only; (2) those with fatigue and EDS; (3) those with fatigue and depression; (4) those with fatigue, EDS and depression (Fig. 4). Very few patients are in fact sleepy or depressed but not fatigued (7%) (Fig. 2). This phenotyping might help in clinical practice: stimulants are preferable in case of fatigue and somnolence without depression (phenotype 2), while antidepressants should be considered if fatigue is only accompanied by bad mood (phenotype 3). However, it remains hard to manage those patients with fatigue alone, i.e. not affected by neither depression nor EDS (phenotype 1; ~ 20%), and those in whom the three symptoms are concomitant (phenotype 4; ~ 17%). In the latter situation, a test of vigilance is recommended to better distinguish sleepiness from depression and chose the proper therapy. The diagnostic diagram proposed in Fig. 4 allows to categorize in a specific sub-group every patient who reaches the outpatient clinic suffering from fatigue.

This study has some limitations. First of all, the exclusion of subjects with an EDSS ≥ 7 and the relatively low median EDSS (2.6) in this MS population limits the generalizability of our results. The absence of an age/sex-matched control group, to compare symptoms’ overlap/correlations between MS and healthy subjects. The linear multiple regression included only fatigue, sleepiness and depression, without considering other possible confounders like sleep disturbances (especially RLS) and symptomatic therapy. According to cross-sectional design of this study we could not conclude about the causality direction among fatigue and depression/sleepiness in MS patients. Finally, the small sample size decreases the statistical power and, therefore, reduces the possibility of reliably characterizing each sub-group.

In conclusion, there is a remarkable overlap between fatigue, depression, and EDS, which requires further research to understand the causality and the physiology behind the demonstrated associations, and to evaluate the effects of specific symptomatic treatment. New trials may profit from the proposed phenotyping.

## Data Availability

The data that support the findings of this study are available from the corresponding author, upon reasonable request.

## Declarations

### Standard protocol approvals, registrations, and patient consents

According to the regulatory requirements of Switzerland, the protocol of the study was approved by the local Independent Ethics Committee (IEC): Comitato etico cantonale (Bellinzona, 6501 Switzerland). Eligible patients signed a written informed consent to participate to the study.
